# A Multinuclear NMR Study on the Speciation in the Liquid-Phase Synthesis of Sulfide-Based Electrolytes for All-Solid-State Lithium Batteries

**DOI:** 10.1021/acs.inorgchem.5c02111

**Published:** 2025-08-26

**Authors:** Agnese Purgatorio, Federico Ducale, Leonardo Tensi, Luca Rocchigiani, Maurizio Leonardi, Alceo Macchioni

**Affiliations:** a Department of Chemistry, Biology and Biotechnology and CIRCC, 9309 University of Perugia, Via Elce di Sotto 8, Perugia 06123, Italy; b Italmatch Chemicals S.p.A., Via S. Tommaso, 13, Spoleto, PG 06049, Italy; c Department of Pharmaceutical Sciences, 9309 University of Perugia, Via del Liceo 1, Perugia 06123, Italy

## Abstract

The preparation of Li_2_S–P_4_S_10_ (LPS) solid electrolytes for all-solid-state lithium batteries through liquid-phase methods has attracted much attention over the last years. One of the most critical aspects to be clarified is how speciation affects the ionic conductivity of the synthesized materials, which is often lower than that obtained by other synthetic approaches. This paper shed some light on the species formed upon the reaction of Li_2_S and P_4_S_10_ in acetonitrile under mild conditions, exploring a wide range of Li_2_S/P_4_S_10_ molar ratios, by means of multinuclear/multidimensional NMR spectroscopy and single-crystal X-ray diffraction. Specifically, ^31^P NMR spectroscopy clearly evidenced the initial formation of P_4_S_11_^2–^ (two triplets at 91.2 and 74.3 ppm, ^2^ *J*_PP_ = 23.16 Hz) from the reaction of P_4_S_10_, solubilized likely via electrostatic interactions (singlet at 81.9 ppm), with Li_2_S. On the way to the LPS solid electrolyte, the twisted (118.3 ppm) and chair (54.3 ppm) isomers of P_2_S_8_^2–^, as well as (PS_3_^–^)_*n*_ (86.5 ppm) and the solvent adduct [PS_3_(CH_3_CN)]^−^ (299.0 ppm), were intercepted. As for (PS_3_^–^)_*n*_, the observation of a sharp singlet in the ^31^P NMR spectrum and the evaluation by diffusion ^31^P NMR spectroscopy of a hydrodynamic volume of ca. 800 Å^3^, substantially the same than that of P_4_S_11_^2–^, suggest the formation of a cyclic P_4_S_12_^4–^ species. Consistently, also P_2_S_6_^2–^, potentially derived by the dissociation in two equal parts of P_4_S_12_^4–^, was observed in the ^31^P NMR spectrum as a singlet at 31.2 ppm. For all the investigated molar ratios, rather broad resonances in the −3.0/–1.5 ppm range were observed in the ^7^Li NMR spectra, reflecting the dynamic nature of these systems. Metathetic exchange of Li^+^ with the bis­(triphenylphosphine)­iminium cation was found to be a successful strategy to obtain good-quality single crystals of P_4_S_11_^2–^, chair and twisted P_2_S_8_^2–^, and P_2_S_6_^2–^, whose solid-state structures were determined by X-ray diffractometric studies.

## Graphical abstract

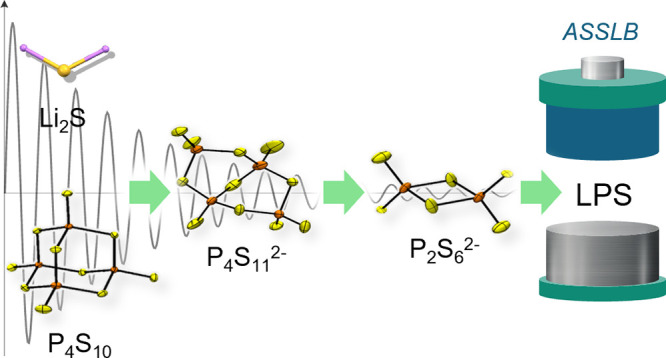

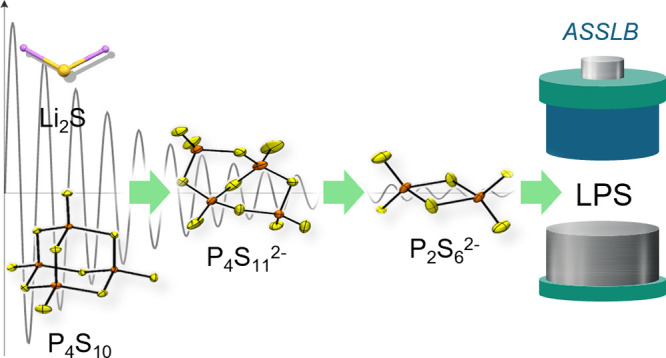

## Introduction

Batteries are poised to play an important role in the transition of modern society toward clean energy solutions and sustainably fulfill the world’s energy demand.^1,2^ In order to meet such goals, accumulators must adapt to stringent performance and safety regulations.^3,4^ All-solid-state lithium-ion batteries (ASSLBs) have recently drawn much attention as they provide enhanced safety with respect to traditional systems owing to their intrinsic reduced flammability. At the same time, they can achieve higher energy densities and longer cycle lifetimes, making them one of the more promising energy storage technologies for electronics and electric vehicles.^5,6^ Several materials have been tested in the recent years, each offering unique advantages and facing specific challenges.^7–10^ Among them, sulfide-based materials have demonstrated significant potential compared to oxide-based and polymeric solid electrolytes, showing high ionic conductivities (up to 12 × 10^–2^ S cm^–1^) and adequate mechanical properties.^11,12^ In the class of sulfide electrolytes, ranging from thio-LISICONs and argyrodites (LPSX) to glass and glass-ceramic materials,^13,14^ binary sulfides with general formula *x*Li_2_S·(100 – *x*)­P_2_S_5_ (also known as LPS systems) are notably interesting since they attain ionic conductivities comparable to those of liquid electrolytes despite having a rather simple composition.^15^

Currently, ball-milling, solid-state, and liquid-phase processes are the most widely used synthetic approaches for the preparation of LPS systems.^16^ In particular, the liquid-phase method has been proposed as an effective route for industrial scaling-up, which would provide homogeneous products avoiding time-intensive and energy-consuming solid-state processes.^17,18^ However, the conductivity of electrolytes synthesized through liquid-phase methods is often lower than those prepared through solid-state procedures; this has been attributed to a slightly different structure, particle morphology, and purity of the resulting materials.^19,20^

To grasp how the wet-chemical approach can be fine-tuned to produce highly conductive solid electrolytes, the understanding of the speciation/aggregation phenomena that occur in solution is crucial, as it might offer a guidance for the rationalization of structure/properties relationship not only for LPS but also for more complex systems such as LGPS (Li_10_GeP_2_S_12_) and LPSX materials.^11^ Several challenges related to the complexity of solution-phase reactions have hampered the definition of a clear reaction mechanism for electrolyte formation in solution, and only few pathways have been proposed so far.^21,22^ In this regard, Calpa et al.^21^ delved into the process of how basic thiophosphate anions, such as P_2_S_6_^2–^, PS_4_^3–^, and P_2_S_7_^4–^, are formed by employing a liquid-phase synthesis in acetonitrile.

According to the proposed mechanism, the preparation of these thiophosphate units starts with the dissociation of P_4_S_10_ into the more reactive P_2_S_5_, which quickly reacts with Li_2_S yielding (PS_3_^–^)_*n*_ chain structures (path **1a** in Figure 1). These chains remain stable up to 180 °C; however, when subjected to thermal treatment above 220 °C, they transform into the less conductive metathiodiphosphate P_2_S_6_^2–^ anions (**2a** in Figure 1). Conversely, when the content of Li_2_S is higher than 50% mol, the formation of PS_4_^3–^ and P_2_S_7_^4–^ units occurs, leading to a much more conductive material. Particularly, when Li_2_S amounts to 70–75% mol (**2b** in Figure 1), the PS_3_^–^ chains that are formed initially incorporate additional Li_2_S and get disrupted upon P–S–P bond cleavage, leading to isolated PS_4_^3–^ units, whereas in the range between 50 and 75% mol of Li_2_S (**1b** in Figure 1), there is not enough sulfur available to fully cleave all the P–S–P bridges in the PS_3_^–^ chains. Consequently, PS_4_^3–^ units and remaining PS_3_^–^ chains coexist in the mixture. Upon heating, the latter undergoes further reaction, leading to the formation of P_2_S_7_^4–^ units (**3** in Figure 1).

**Figure 1:**
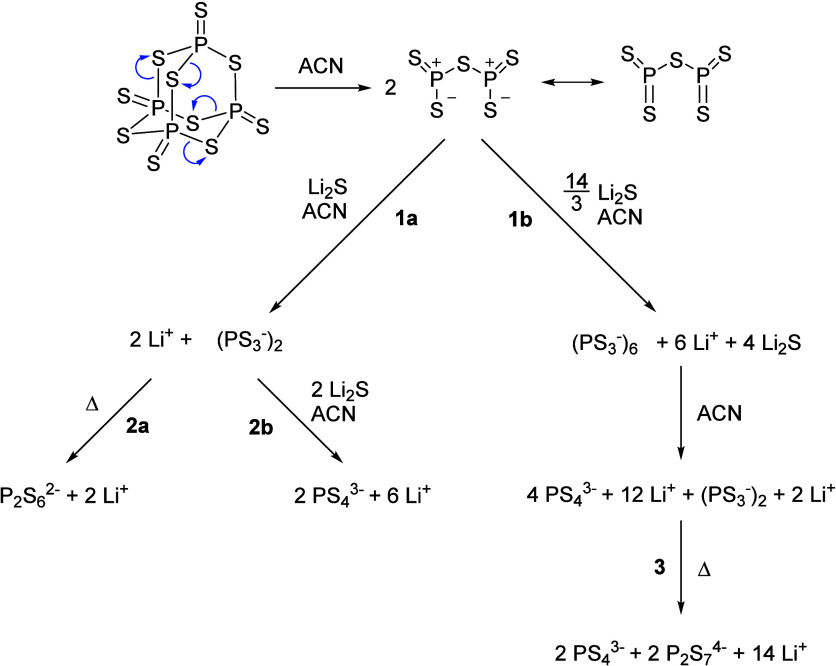
Reaction pathways proposed by Calpa et al. where the generation of PS_3_^–^ chain units (**1a** and **1b**) represents the initial step for the formation of metathiodiphosphate P_2_S_6_^2–^ (**2a**), orthothiophosphate PS_4_^3–^ (**2b**), and pyrothiophosphate P_2_S_7_^4–^ anions (**3**).^21^

Interestingly, findings by Wang et al. on the synthesis of Li_7_P_3_S_11_ complement Calpa’s results, providing a more complex mechanism involving the formation of additional intermediates.^22^ Indeed, Li_2_P_4_S_11_ is proposed as the first intermediate for the reaction between Li_2_S and P_2_S_5_ in a 1:2 molar ratio (path **1** in Figure 2). Seemingly, a nucleophilic attack by the lone pair of electrons on the sulfur atom in Li_2_S targets a P–S bond in the adamantane-like P_4_S_10_ structure, partially opening the cage, and giving rise to Li_2_P_4_S_11_. A second nucleophilic attack from Li_2_S further opens the structure (**2** in Figure 2), leading to the formation of Li_4_P_4_S_12_. Additional Li_2_S (path **3**) then separates insoluble Li_3_PS_4_ from Li_4_P_2_S_7_, which remains in solution. The final step (**4** in Figure 2) involves solvent removal and annealing, prompting the crystallization of Li_7_P_3_S_11_.

**Figure 2:**
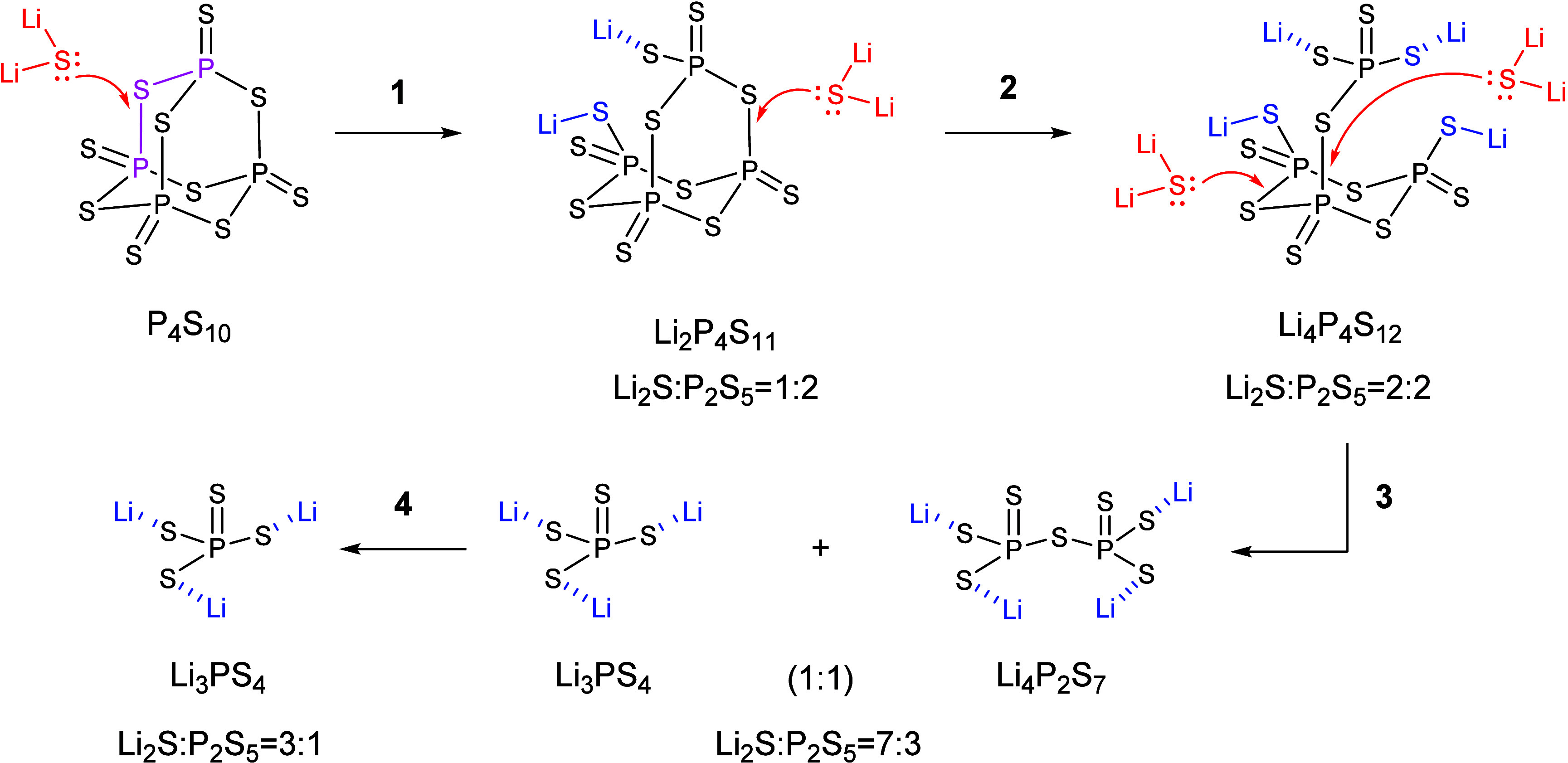
Reaction mechanism proposed by Wang et al. of different ratios of Li_2_S and P_2_S_5_ in acetonitrile. Adapted with permission from Wang et al., 2020.^22^ Copyright [2025] [American Chemical Society].

It is also important to recall the results by Delnick et al., which provide convincing evidence, obtained mainly by Raman spectroscopy, of the crucial role of a lithitropic tautomer of P_2_S_6_^2–^ in mediating the reaction of Li_2_S and P_2_S_5_ in several solvents.^23^

The results reported above underline the molecular complexity related to the synthesis of thiophosphates and emphasize the need for further investigations of the roles of various intermediates in tailoring the properties of solid electrolytes starting from the processes that occur in solution.

We decided to study in-depth the thiophosphate synthesis by means of multinuclear and diffusion NMR spectroscopic techniques, which are powerful tools already successfully applied for investigating the reactivity of complex inorganic/organometallic systems, as well as their aggregation in solution,^24^ and weakly interacting systems^25,26^ and ion pairing phenomena^24,27^ relevant to organometallic catalysis^28,29^ and materials chemistry.^27^ Particularly, herein, we report the results of a systematic ^1^H, ^7^Li, and ^31^P NMR study on the reactivity of Li_2_S and P_4_S_10_ carried out under mild conditions (room temperature), deliberately and systematically changing their stoichiometric molar ratio from 0.125:1 to 6:1, in order to intercept the intermediates of the early stage of LPS synthesis. The obtained results were complemented by single-crystal XRD analysis to sketch out a complete reaction pathway for the formation of the LPS system in solution. Single crystals of suitable quality for X-ray diffractometry studies were obtained for many intermediates only by a metathetic exchange of Li^+^ with a much bigger cation as bis­(triphenylphosphine)­iminium (PPN^+^).

## Experimental Section

### General

Due to the sensitivity of sulfides to atmospheric moisture, all starting materials and samples were handled in a nitrogen-filled glovebox. Li_2_S (Sigma-Aldrich, 99.98%) and P_4_S_10_ (Italmatch Chemicals, content of P equal to 27.87%, with 0.01% of free sulfur) were used without further purification. CS_2_ (Sigma-Aldrich, ≥99.9%) was dried over 4 Å molecular sieves, while toluene (Sigma-Aldrich, ≥99.5%) and acetonitrile-*d*_*3*_ (Euriso-TOP, ≥99.80 atom % D) were distilled over Na/K alloy and CaH_2_, respectively.

### Sample Preparations

CS_2_ or toluene was added to P_4_S_10_. The mixtures were stirred for 3 h until P_4_S_10_ was completely solubilized, obtaining a colorless solution (4 mM).

As far as LPS intermediates are concerned, the typical experimental procedure consisted of charging Li_2_S and P_4_S_10_ in different molar ratios into a scintillation vial equipped with a magnetic stirrer (Table 1). A 1 mL portion of acetonitrile-*d*_3_ was added to the powders at room temperature, and the mixture was stirred for 3 h. The resultant suspensions were filtered over a Kimwipe to obtain clear solutions.

**Table 1:** Details of the Experimental Procedure for Preparing Li_2_S–P_4_S_10_ Samples in Acetonitrile

Li_2_S:P_4_S_10_ (molar ratio)	Li_2_S (mg, mmol)	P_4_S_10_ (mg, mmol)	mixture appearance (supernatant, precipitate)
0.125:1	0.6, 0.013	46.9, 0.105	pale-yellow, yellow
0.25:1	1.2, 0.026	45.3, 0.102	pale-yellow, yellow
0.5:1	2.5, 0.054	47.1, 0.106	pale-yellow, yellow
1:1	4.8, 0.104	46.4, 0.104	yellow, white
2:1	8.6, 0.187	41.4, 0.093	yellow, white
4:1	14.7, 0.320	35.3, 0.079	yellow, white
14:3	17.4, 0.379	36.3, 0.082	yellow, white
6:1	20.6, 0.448	33.0, 0.074	colorless, white

### NMR Spectroscopy

NMR spectra were recorded on a Bruker Avance III 400 spectrometer equipped with a smartprobe (400.13 MHz for ^1^H) with a *z* gradient coil or on a Bruker Avance NEO 600 spectrometer equipped with the Prodigy Bruker CryoProbe (600.13 MHz for ^1^H) with a *z* gradient coil. Residual solvent resonances of acetonitrile-*d*_3_ were used for referencing the ^1^H spectra. Chemical shifts are relative to external 85% H_3_PO_4_ for ^31^P (161.923 MHz for the 400.13 MHz spectrometer, and 242.884 MHz for the 600.13 MHz spectrometer) and LiCl 9.7 m in D_2_O for ^7^Li (155.454 MHz for the 400.13 MHz spectrometer, and 233.181 MHz for the 600.13 MHz spectrometer).

^1^H, ^7^Li, and ^31^P PGSTE (Pulsed Gradient STimulated Echo) NMR measurements were performed at 298 K by using the standard double-stimulated echo pulse sequence (dstegp3s1d) without spinning. The shape of the gradients was rectangular, and their strength was varied during the experiments. According to eq [Disp-formula eqa], the translational self-diffusion coefficient (*D*_t_) was calculated from the slope of ln­(*I*/*I*_0_) versus *G* ^2^ trends, in which *I* and *I*_0_ are the intensity of the resonance in the presence and in the absence of the pulsed-field-gradient, *G* is the gradient strength, Δ is the delay between the midpoints of the gradients, and δ is the gradient length.^30,31^

$$\ln\frac{I}{I_{0}}=-{\left(\gamma \delta \right)}^{2}G^{2}\left(\Delta -\frac{\delta }{3}\right)D_{t}$$ a

The hydrodynamic radius (*r*_H_) was calculated from *D*_t_ by using the Stokes–Einstein equation (eq [Disp-formula eqb]), where *k*_B_ is the Boltzmann constant, *T* is the temperature in Kelvin, η is the viscosity of the solution, and *c* is a numerical factor that accounts for the ratio between the *r*_H_ of the analyte and that of the solvent.^30^

$$D_{t}=\frac{k_{B}T}{c\pi \eta r_{H}}$$ b

PGSTE NMR experiments for P_4_S_10_ were carried out in CS_2_ by using benzene (2 μL, 0.022 mmol) as an internal standard or in toluene with an acetone-*d*_*6*_ capillary, while acetonitrile-*d*_3_ was employed for the investigation in solution of the LPS intermediates.

In all PGSTE measurements, δ = 2.5 ms, Δ = 200–400 ms, while gradient recovery delays (d16) were 200 μs. The number of scans varied between 8 and 32 per increment per ^1^H and ^7^Li PGSTE, while between 512 and 1024 per increment per ^31^P PGSTE.

For the estimation of longitudinal relaxation times *T*_1_, a standard inversion recovery (t1ir) pulse sequence was used.^32–34^

### XRD Analysis

X-ray diffraction patterns of single crystals were recorded at low temperature by using a Bruker D8 Venture diffractometer equipped with an Incoatec ImuS3.0 microfocus sealed-tube Mo Kα (λ = 0.71073 Å) source and a CCD Photon II detector. The data, collected through generic φ and ω scans, were integrated and reduced using the Bruker AXS V8 Saint Software. The structure was solved and anisotropically refined using the SHELXT and SHELXL packages of the Bruker APEX3 software.^35^

## Results and Discussion

P_4_S_10_ alone was studied to have a reference for the subsequent reactions with Li_2_S performing longitudinal relaxation times measurements and diffusion NMR studies in two different nonpolar solvents, namely, carbon disulfide and toluene. The latter were selected due to the insolubility of P_4_S_10_ in polar media, such as acetonitrile, which was used instead in the following reaction steps. These studies were intended to complement ^31^P NMR data already reported in the literature.^36^

A singlet at 55.9 ppm, corresponding to P_4_S_10_, and a complex system of resonances ranging from 63.1 to 56.0 ppm, due to the second order AB_3_ spin system of P_4_S_9_, are visible in the ^31^P NMR spectra (Figures S1 and S2 in the Supporting Information) in both solvents.^36^ The longitudinal relaxation times (*T*_1_) of P_4_S_10_ and P_4_S_9_ in CS_2_ and toluene were measured by means of ^31^P inversion recovery experiments (a–d in Figure S5). In carbon disulfide, *T*_1_ values are 11 s for P_4_S_9_ and 13 s for P_4_S_10_, while much shorter *T*_1_ values are measured in toluene (*T*_1_ = 5 s), probably due to intermolecular dipolar P–H interactions with the protons of the solvent. PGSTE NMR measurements were conducted to estimate the hydrodynamic dimensions of P_4_S_10_ in solution. The elaboration of the diffusion experiments (Figures S6 and S7) affords a hydrodynamic volume (*V*_H_) equal to 237 ± 36 Å^3^ in CS_2_ and 310 ± 46 Å^3^ in toluene. These values are very close to both the van der Waals volume (*V*_VdW_) of P_4_S_10_, estimated at 250 Å^3^ using an MMFF level of semiempirical calculations, and its crystallographic volume of 346 Å^3^, obtained by the single-crystal XRD analysis of P_4_S_10_ crystals (see XRD Analysis and the Supporting Information (SI) for more details). These findings clearly indicate that P_4_S_10_ is not aggregated in CS_2_ or toluene solutions and likely behaves as a monomeric species.

### Solution-Phase Studies on the Reaction of P_4_S_10_ with Li_2_S

To better understand the step-by-step processes involved in the synthesis of LPS systems in solution, we investigated the reactivity of P_4_S_10_ with Li_2_S in acetonitrile, replicating the procedure that leads to high-quality and well-defined sulfide electrolytes, which are critical for the performance of ASSLBs.^37^ P_4_S_10_ and Li_2_S were mixed at room temperature in different molar ratios, starting with a composition where Li_2_S is substoichiometric with respect to P_4_S_10_ and progressively increasing the amount of Li_2_S to that required to form Li_3_PS_4_. After 3 h under stirring, the obtained mixtures were filtered to remove the residual solid, and the supernatant was analyzed by NMR spectroscopy.

The ^31^P NMR spectrum of the Li_2_S:P_4_S_10_ = 0.125:1 ratio (a in Figure 3) shows the main presence of a singlet at 81.9 ppm and many other resonances of lower intensity.

**Figure 3:**
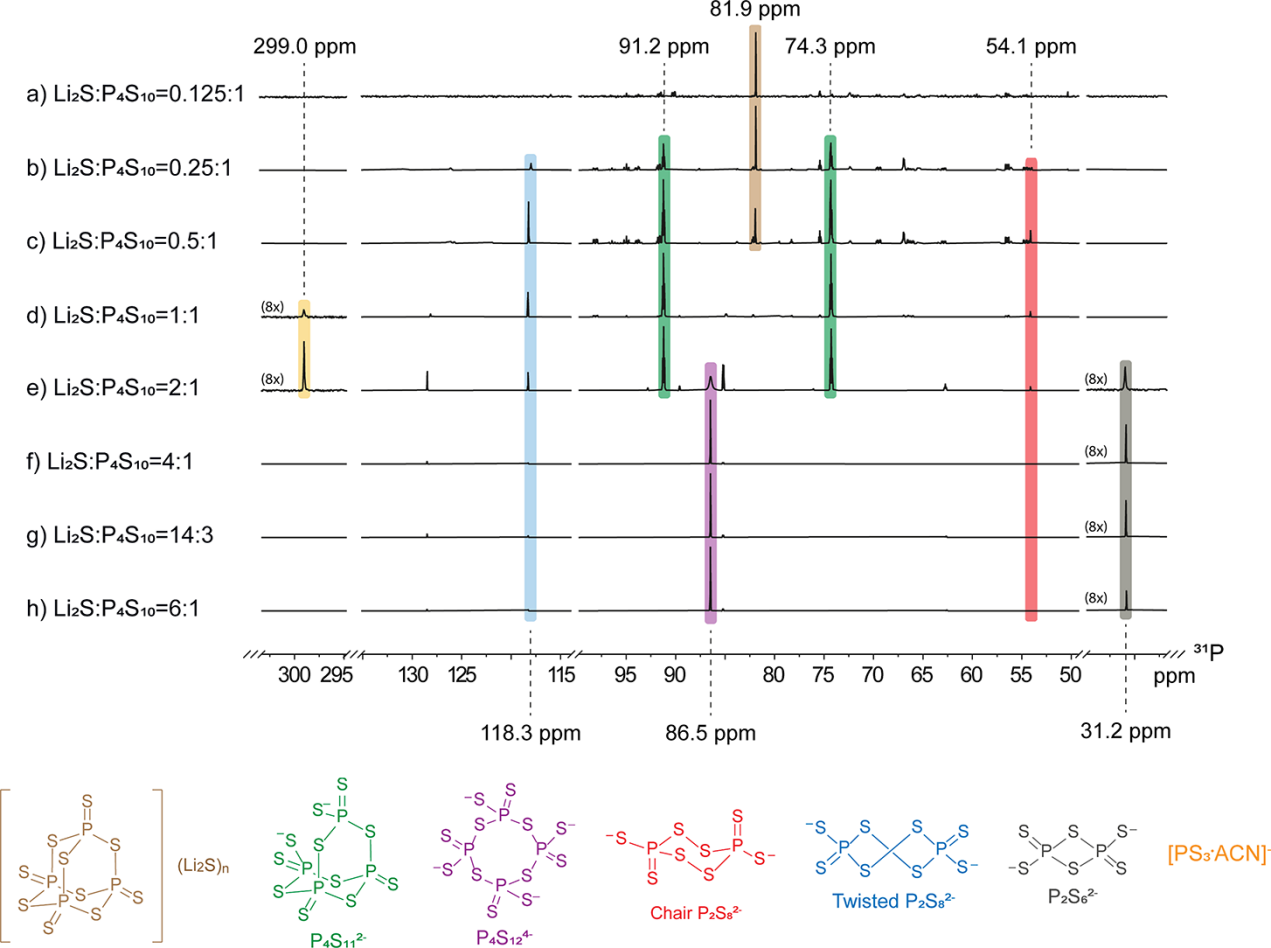
^31^P NMR spectra observed at different Li_2_S:P_4_S_10_ molar ratios.

^31^P PGSTE NMR experiments (Figure S8) on the signal at 81.9 ppm afforded a *V*_H_ value of 359 ± 54 Å^3^ (entry 2 in Table 2), which is superimposable to that of P_4_S_10_. ^7^Li diffusion measurements on the signal at −2.7 ppm (a in Figure S14) yielded a very similar value of *V*_H_ = 369 ± 55 Å^3^ (entry 1 in Table 2). The strong match between the hydrodynamic dimensions obtained from ^31^P and ^7^Li signals suggests that substoichiometric lithium quantitatively interacts with P_4_S_10_ cages, which are still intact in solution. Therefore, we speculate that the resonance at 81.9 ppm, never observed before, likely because previous studies were performed at higher temperature and Li_2_S/P_4_S_10_ molar ratios, might be assigned to a [P_4_S_10_]­(Li_2_S)_*n*_ intermediate mainly held together by electrostatic interactions. The presence of a singlet may be related to a dynamic phenomenon that mediates the ^31^P chemical shift at room temperature. Unfortunately, this species is not thermally stable and could not be isolated.

**Table 2:** *D* (× 10^9^ m^2^ s^–1^), *r*_H_ (Å), and *V*_H_ (Å^3^) Values in Acetonitrile-d_3_ at 298 K^a^

entry	Li_2_S:P_4_S_10_	nucleus	δ (ppm)	*D*	*r*_H_	*V*_H_
1	0.125:1	^7^Li	–2.7	1.56 ± 0.08	4.45	369 ± 55
2		^31^P	81.9	1.57 ± 0.08	4.41	359 ± 54
3	1:1	^7^Li	–2.5	1.55 ± 0.08	4.52	387 ± 58
4		^31^P (P_4_S_11_^2–^)	91.2	1.17 ± 0.06	5.77	804 ± 121
5		^31^P (P_4_S_11_^2–^)	74.3	1.18 ± 0.06	5.72	784 ± 118
6	2:1	^7^Li	–2.0	2.51 ± 0.13	4.57	400 ± 60
7		^31^P (PS_3_^–^)_*n*_	86.5	3.33 ± 0.17	5.8	817 ± 123
8	4:1	^7^Li	–1.5	2.65 ± 0.13	4.79	460 ± 69
9		^31^P (PS_3_^–^)_*n*_	86.5	2.32 ± 0.12	5.84	834 ± 125
10	14:3	^7^Li	–1.5	2.53 ± 0.13	4.61	410 ± 62
11		^31^P (PS_3_^–^)_*n*_	86.5	3.25 ± 0.16	5.73	788 ± 118
12	6:1	^7^Li	–1.4	3.33 ± 0.17	4.75	449 ± 67
13		^31^P (PS_3_^–^)_*n*_	86.5	2.63 ± 0.13	5.85	838 ± 126

a A 5% experimental error is assumed in *D*_t_, which implies 15% uncertainty in *V*_H_.^30,31^

Upon doubling the amount of Li_2_S with respect to P_4_S_10_ (Li_2_S:P_4_S_10_ = 0.25:1, **3b**), the formation of two triplets at 91.2 and 74.3 ppm (^2^ *J*_PP_ = 23.16 Hz) was observed in the ^31^P NMR spectrum. Interestingly, these signals grow in intensity by further increasing the Li_2_S:P_4_S_10_ ratio, reaching a maximum at the 1:2 molar ratio, where they become the dominant signals (panel **d** in Figure 3, with an expanded view displayed in **a** in Figure 4). ^31^P-COSY NMR shown in Figure 4b confirms that the two triplets are scalarly coupled forming an A_2_X_2_ spin system.

**Figure 4:**
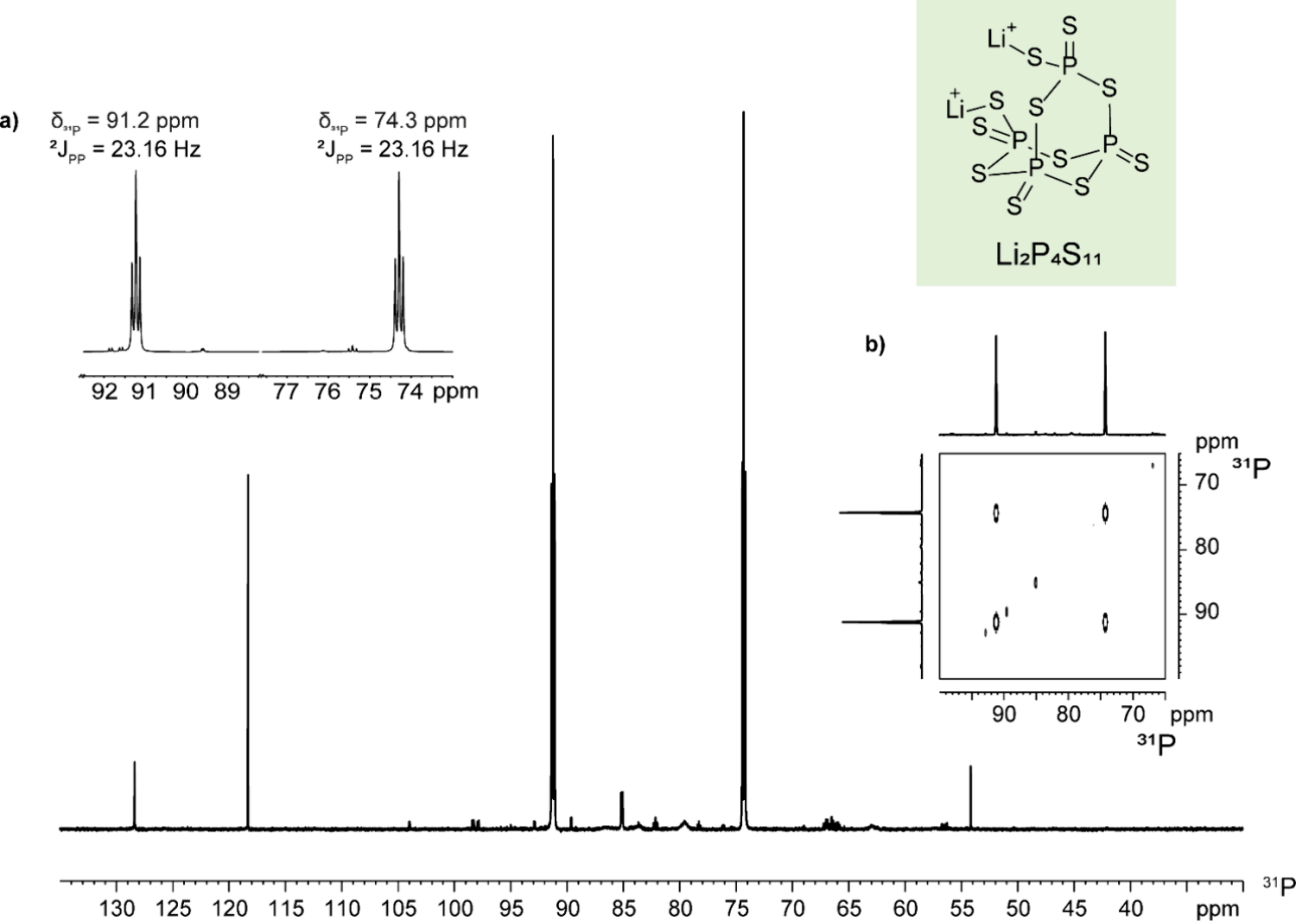
(a) ^31^P NMR spectrum of the Li_2_S:P_4_S_10_ = 1:1 molar ratio, zooming on the two triplets at 91.2 and 74.3 ppm. (b) Section of the ^31^P-COSY NMR spectrum of the same sample (CD_3_CN, 298 K).

Based on such observations, this intermediate can be assigned to Li_2_P_4_S_11_, which formally derives from the addition of a Li_2_S molecule to a P_4_S_10_ cage. Most likely, the formation of terminal P–S–Li moieties is responsible for the low-frequency shift of the resonance at 74.3 ppm, while the other two phosphorus atoms are less affected and remain at higher frequencies (91.2 ppm).

A series of PGSTE NMR measurements has been performed on Li_2_P_4_S_11_ (Figure 5), affording a *V*_H_ of 804 ± 121 Å^3^ and 784 ± 118 Å^3^ (entries 4 and 5 in Table 2, respectively) for the two triplets at 91.2 and 74.3 ppm, respectively. On the other hand, ^7^Li diffusional measurements on the signal at −2.5 ppm (**d** in Figure S14) resulted in a *V*_H_ of 387 ± 58 Å^3^ (entry 3 in Table 2).

**Figure 5:**
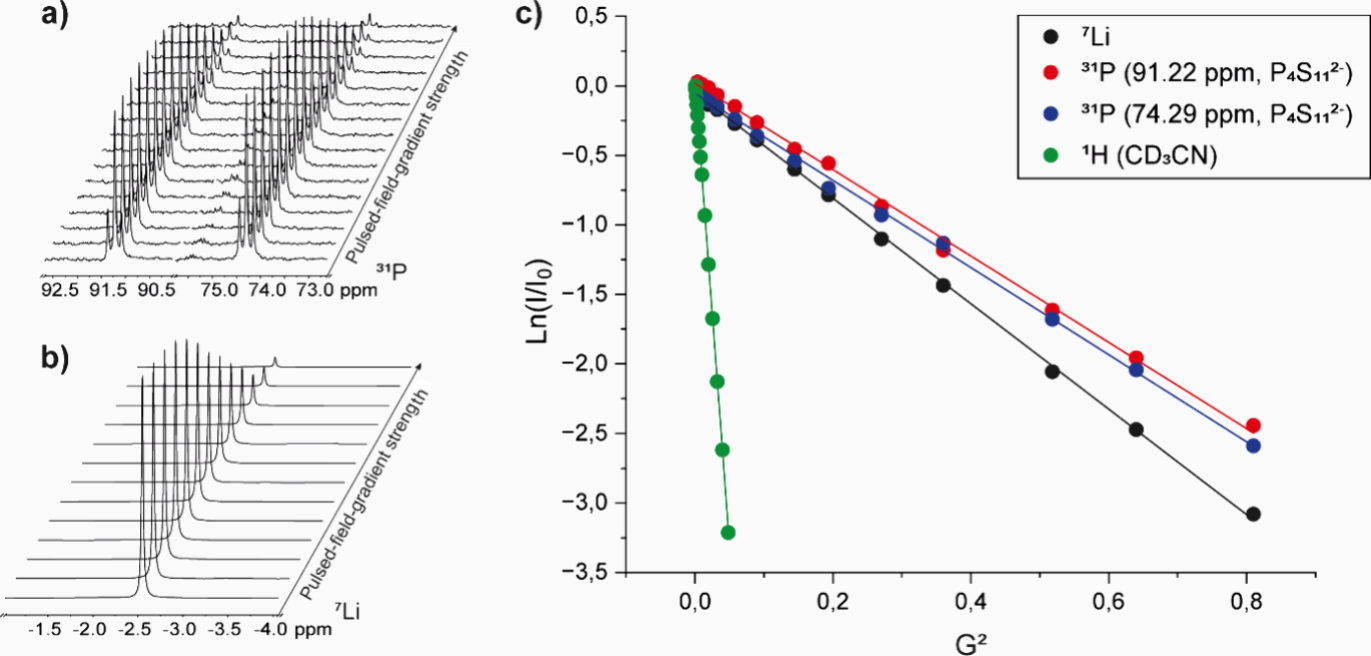
Monodimensional decay of the intensity of (a) ^31^P and (b) ^7^Li resonances of P_4_S_11_^2–^ in acetonitrile-d_3_ obtained by PGSTE NMR. (c) Trends of ln­(*I*/*I*_0_) vs G^2^ of P_4_S_11_^2–^.

Given that monomeric P_4_S_11_^2–^ and P_4_S_10_ are expected to be comparable in size, the increased *V*_H_ values observed by ^31^P PGSTE NMR for the former (Table 2) suggest that P_4_S_11_^2–^ undergoes some self-aggregation in solution. On the other hand, the apparent *V*_H_ value for the cationic Li species is lower than that of P anionic ones, suggesting that ion pairing is not quantitative, and a fraction of Li ions is “freely” diffusing in solution. A ^31^P inversion recovery experiment afforded a *T*_1_ of 4 s for these signals, with the corresponding plot displayed in **e** in Figure S5.

Although the formation of P_4_S_11_^2–^ had been already hypothesized by Wang et al. for the solution synthesis of Li_7_P_3_S_11_,^22^ it has not been previously observed likely because precedent experiments have been conducted in the presence of a larger quantity of Li_2_S and at higher temperatures. In this work, we provide the first direct observation and complete characterization of such species also in the solid state (*vide infra*).

The resonances observed at 118.3 and 54.3 ppm in the ^31^P NMR spectra recorded for the Li_2_S:P_4_S_10_ solution from 0.25:1 to 6:1 (Figure 3b–h) are attributed to the twisted and chair isomers of P_2_S_8_^2–^, respectively.^38^ The formation of the latter suggests that redox processes take place in solution, as the formation of disulfur bridges requires a formal oxidation of sulfur atoms from −2 to −1. It is reasonable that these processes are triggered by the presence of some S_8_, which is a known contaminant of commercial samples of P_4_S_10_, detected at a concentration of 0.01%.

The NMR spectra of the Li_2_S:P_4_S_10_ = 1:1 and Li_2_S:P_4_S_10_ = 2:1 mixtures (Figure 3d,e) also show the presence of a minor singlet at high frequencies (δ_P_ = 299.0 ppm), which disappears at higher molar ratios. Rotter previously reported a similar signal at 297.5 ppm for the PS_3_^–^ species with a coordinated pyridine molecule.^38^ Based on this experimental observation, we hypothesize that the observed signal at 299.0 ppm may belong to the [PS_3_(CH_3_CN)]^−^ solvent adduct. At higher Li_2_S:P_4_S_10_ mole ratios (e in Figure 3), a singlet at 31.2 ppm appeared, which was previously assigned to the P_2_S_6_^2–^ species.^38^ Such observations suggest that at lower PS_3_^–^ concentrations, the monomeric [PS_3_(CH_3_CN)]^−^ form predominates, while at higher concentrations dimerization can occur, leading to the formation of P_2_S_6_^2–^ units.

When the amount of Li_2_S was further increased with respect to P_4_S_10_ (e–h in Figure 3), the resonances associated with P_4_S_11_^2–^ decreased in intensity, and an intense sharp singlet appeared at 86.5 ppm. This resonance has been previously attributed in the literature to the polymeric (PS_3_^–^)_*n*_ species.^21^ By performing a series of ^31^P PGSTE NMR at molar ratios ranging from Li_2_S:P_4_S_10_ = 2:1 to Li_2_S:P_4_S_10_ = 6:1, the resonance at 86.5 ppm affords a *V*_H_ of about 819 Å^3^ (by averaging entries 7, 9, 11, and 13 in Table 2). Using the MMFF level of semiempirical calculations, the *V*_VdW_ of a singlet PS_3_^–^ unit was estimated to be 89 Å^3^. *V*_H_ is usually ca. 1.5 times *V*_VdW_, suggesting that the polymeric chain of PS_3_^–^ consists of a maximum of six PS_3_^–^ units, assuming that self-aggregation is not present. Nevertheless, it is important to outline that the average *V*_H_ of (PS_3_^–^)_*n*_ is substantially the same than that of P_4_S_11_^2–^. Furthermore, a linear structure of (PS_3_^–^)_*n*_ with *n* < 6 should exhibit inequivalent and structured ^31^P NMR resonances. The observation of singlet and a *V*_H_ comparable to that of P_4_S_11_^2–^ induces us to propose that the species associated with the resonance at 86.5 ppm is cyclic (PS_3_^–^)_4_, which, being multianionic, undergoes some self-aggregation in acetonitrile. On the other hand, diffusion measurements on ^7^Li nucleus showed an average *V*_H_ of 430 Å^3^ (by averaging entries 6, 8, 10, and 12 in Table 2), indicating that, as observed for P_4_S_11_^2–^, an equilibrium between free and associated lithium ions is present. Notably, cyclic (PS_3_^–^)_4_ has already been observed in both the two-(2D) and three-dimensional (3D) forms of NbP_2_S_8_;^39^ however, all attempts to obtain single crystals or a pure sample of the compound failed, thereby precluding complete characterization.

Monodimensional ^7^Li NMR spectra, acquired for all stoichiometric ratios (Figure S14), show only a moderate shift of the singlet from about −3.0 to approximately −1.5 ppm, despite the complexity of the reactions. Consequently, they are little informative on the structure of the salts obtained.

### Single-Crystal XRD

To better understand the molecular nature of the thiophosphate species formed by the reaction of P_4_S_10_ with Li_2_S, single-crystal X-ray diffraction studies were carried out.

Single crystals of P_4_S_10_ suitable for X-ray analysis were obtained by precipitation from a saturated solution in CS_2_. As already reported in the literature,^40^ P_4_S_10_ crystallizes with a triclinic P-1 unit cell and it features the classical adamantane-like structure. All the P atoms of the cage are characterized by a tetrahedral geometry (S–P–S bond angles ranging from 108.30° up to 110.53°), and as expected, they display shorter bonds with the terminal sulfur (1.9052(7)–1.9125(5) Å) with respect to the bridging ones (2.0926(4)–2.1015(5) Å).

All of the attempts to crystallize ionic lithium thiophosphates resulted unsuccessful. A strategy to obtain crystals of good quality for single-crystal X-ray diffraction studies was found considering that ionic compounds tend to form more stable solid-state structures when cation and anion are comparable in size.^41^ For such a reason, bis­(triphenylphosphine)­iminium chloride ([PPN]­[Cl]) was added to the reaction mixture in order to exchange Li^+^ with the bulkier PPN^+^ cation.

The strategy revealed to be successful, since the addition of [PPN]­[Cl] in the Li_2_S:P_4_S_10_ = 1:1 mixture resulted in the precipitation of yellow prismatic crystals, suitable for X-ray diffraction studies (Tables S1 and S2). Solution and refinement yielded the structure reported in Figure S15, in which a molecule of [P_4_S_11_]­[PPN]_2_ cocrystallized with either [P_2_S_8_]­[PPN]_2_ (crystallographic occupancy ca. 0.9) or [P_2_S_6_]­[PPN]_2_ (crystallographic occupancy ca. 0.1). P_4_S_11_^2–^ anion (Figure 6a) crystallized with two possible orientations in a 50:50 ratio roto-reflected of about 90° between each other.

**Figure 6:**
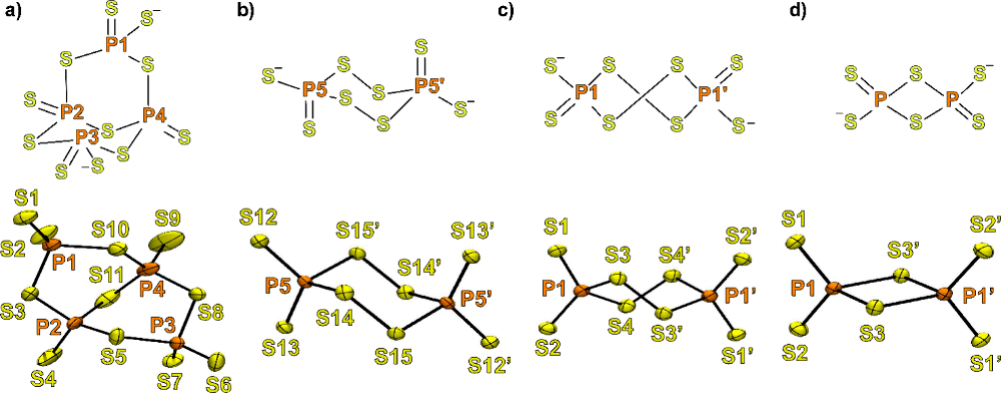
ChemDraw and Ortep drawings of crystal structures of (a) P_4_S_11_^2–^, (b) chair, and (c) twisted P_2_S_8_^2–^, and (d) P_2_S_6_^2–^ (PPN cations are omitted for clarity) obtained by single-crystal X-ray diffraction. Ellipsoids are drawn at the 50% probability level. For further details, see the SI.

It features an inner core characterized by an eight-membered ring, formed by four phosphorus and four chalcogen atoms alternating with each other, split into two halves by the presence of a bridging sulfur (S11) connecting P2 and P4 (Figure 6a) and structurally resembling a byciclo[3.3.1]­nonane. While P2 and P4 display a single terminal chalcogen atom, as in the case of P_4_S_10_, P1 and P3 are characterized by the presence of two terminal sulfur atoms that heavily affect the tetrahedral geometry of the two phosphorus atoms (S1–P1–S2 = 121.1(1)°, S3–P1–S10 = 99.65(9)°, S6–P3–S7 = 122.7(1)°, and S5–P3–S8 = 98.79(8)°). Such difference is also reflected in the bond lengths between the P atoms and the bridging S. Indeed, P1 and P3 display longer bonds (P1–S3 = 2.151(2) Å, P1–S9 = 2.192(2) Å, P3–S5 = 2.178(2) Å, and P3–S8 = 2.149(2) Å) with respect to P2 and P4 (P2–S3 = 2.085(3) Å, P2–S5 = 2.088(3) Å, P4–S8 = 2.069(2) Å, and P4–S10 = 2.061(3) Å). Additionally, the P2–S11 (2.116(3) Å) and P4–S11 (2.114(3) Å) bonds are slightly longer with respect to P2–S3, P2–S5, P4–S8, and P4–S10 probably due to the steric tension of the eight-membered ring.

The bonds between the phosphorus and the terminal sulfur atoms in the P_4_S_11_^2–^ anion are generally longer (P1–S1 = 1.947(2) Å, P1–S2 = 1.936(2) Å, P2–S4 = 1.929(4) Å, P3–S6 = 1.961(2) Å, P3–S7 = 1.929(2) Å, and P4–S10 = 1.930(2) Å) with respect to the ones of P_4_S_10_.

As mentioned above, together with [P_4_S_11_]­[PPN]_2_, also molecules of [P_2_S_8_]­[PPN]_2_ and [P_2_S_6_]­[PPN]_2_ are present in the crystal structure. High-quality SC-XRD structures of the [P_2_S_8_]­[PPN]_2_ ion pair (Figure 6b) were obtained. The latter displays a core featuring a six-membered ring in the classic chair conformation with the two PS_2_^–^ moieties in positions 1 and 4 pointing in opposite directions. From the structural point of view, all the bond lengths and angles are in line with what is already observed in analogous structures characterized by different cations.^42–44^ Similarly to P_4_S_11_^2–^, the P atoms display a distorted tetrahedral geometry (S12–P5–S13 = 124.6(1)° and S14–P5–S15 = 102.73(6)°) and elongated bonds with the bridging sulfur atoms (P5–S14 = 2.129(1) Å and P5–S15 = 2.133(2) Å). On the other hand, it was not possible to derive reliable structure factors for [P_2_S_6_]­[PPN]_2_ due to its low occupancy (ca. 10 %) and the remaining disorder of the asymmetric unit. However, crystals of pure [P_2_S_6_]­[PPN]_2_ were obtained by precipitation from different reaction mixtures (see below).

A separate crystallization attempt successfully led to [P_2_S_8_]­[PPN]_2_ in its twisted conformation (Figure 6c). Particularly, in this case, the six-membered ring displays a distorted boat conformation with S3 and S4′ atoms that lies on the apical positions. Comparing the structures of the two conformers, while the P atoms of the P_2_S_8_^2–^ in the boat conformation are still characterized by a distorted tetrahedral geometry (S1–P1–S2 = 121.64(7)° and S3–P1–S4 = 102.49(6)°), they display longer bonds (P1–S3 = 2.145(2) Å and P1–S4 = 2.141(2) Å) with bridging sulfur with respect to the “chair” isomer.

Crystallization of the components of the Li_2_S:P_4_S_10_ = 6:1 reaction mixture by addition of [PPN]­[Cl] yielded colorless single crystals of [P_2_S_6_]­[PPN]_2_. The latter species features a dimeric structure involving two PS_3_^–^ moieties, similar to a diborane molecule. The four-membered ring displays an almost perfect square geometry with similar bond lengths (P1–S3 = 2.1318(6) Å and P1–S3′ = 2.1564(6) Å) and angles close to 90° (S3–P1–S3′ = 91.48(2)° and P1–S3–P1′ = 88.52(2)°). As a result, the P atoms are characterized by a strongly distorted tetrahedral geometry in which the angle between the P and the two terminal sulfurs is close to 120° (S1–P1–S2 = 118.25(3)°).

### Proposed Reaction Mechanism for the Formation of the LPS System in Solution

The outcomes of the individual reactions described above can be rationalized as snapshots of the progressive reactivity between Li_2_S and P_4_S_10_ leading to the formation of Li_3_PS_4_ and Li_7_P_3_S_11_ in solution. Therefore, the results obtained from heteronuclear and diffusional NMR experiments, along with XRD analysis, can be used to identify a compelling reaction mechanism for the generation of thiophosphate salts in acetonitrile.

According to our observations, the initial stage of the reaction (path **1** in Figure 7) seems to be the solubilization of P_4_S_10_, probably due to the establishment of electrostatic interactions with lithium sulfide [P_4_S_10_]­(Li_2_S)_*n*_. This is the entry point for P_4_S_10_, which is completely insoluble in acetonitrile. Such a hypothesis is supported by the observation of a single intense signal at 81.9 ppm in the ^31^P NMR spectrum and by a series of ^7^Li and ^31^P PGSTE NMR experiments, which revealed that the *V*_H_ values measured for both the nuclei are similar.

**Figure 7:**
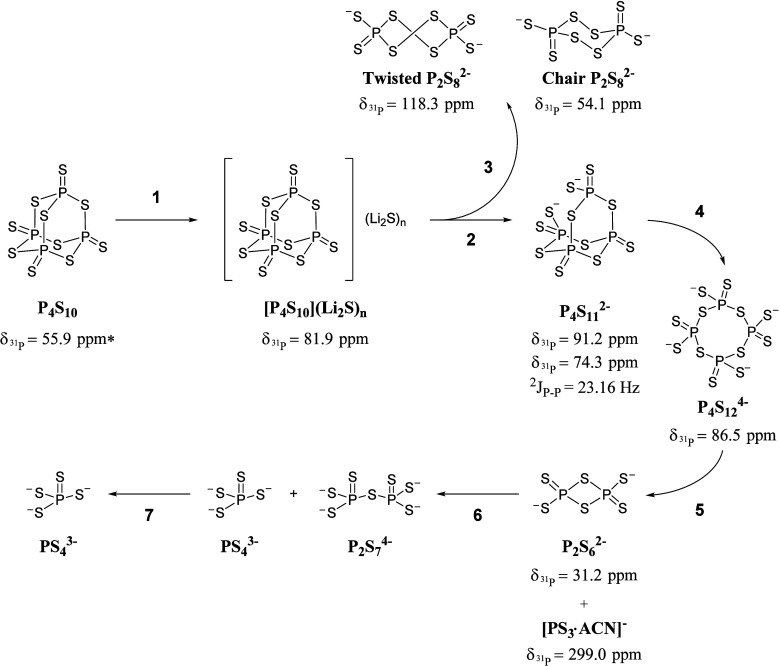
Formation reaction scheme of the pivotal intermediates in the preparation of the LPS system in solution.

As the reaction proceeds (path **2** in Figure 7), the P_4_S_10_ cage is disrupted by Li_2_S leading to the formation of P_4_S_11_^2–^_,_ whose identity was spectroscopically and crystallographically confirmed for the first time. The formation of the latter aligns with the proposal by Wang et al. for the solution synthesis of Li_7_P_3_S_11_, where Li_2_S is thought to act as a nucleophile toward the P–S–P bonds within the P_4_S_10_ cage.^22^ To prove the above mechanism, Li_2_S was substituted with LiCl and reacted with P_4_S_10_ in a LiCl:P_4_S_10_ = 1:1 molar ratio. Surprisingly, the recorded ^31^P NMR spectrum was similar to that obtained for the Li_2_S:P_4_S_10_ = 0.25:1 molar ratio. As shown in Figure S3, the two triplets at 91.2 and 74.3 ppm, corresponding to the P_4_S_11_^2–^ species, are visible along with the signal at 81.8 ppm. While the exact role of LiCl in promoting the formation of P_4_S_11_^2–^ remains uncertain, it is nonetheless surprising that a similar reactivity is observed even in the absence of Li_2_S. It may be speculated, at first, that LiCl and the trace amounts of S_8_ in the P_4_S_10_ sample could potentially generate nucleophilic S^2–^ ions, thus explaining why LiCl and Li_2_S afford the same reactivity. Further experimental and DFT mechanistic studies will be necessary to understand how P_4_S_11_^2–^ forms, a process that evidently involves a reaction pathway more complex than previously proposed.

After cage opening, increasing the amount of Li_2_S leads to a disappearance of P_4_S_11_^2–^ and a gradual formation of the (PS_3_^–^)_*n*_ polymeric species, likely as a cyclic P_4_S_12_^4–^ under mild conditions used by us, which becomes the most abundant anion in solution.

The formation of PS_4_^3–^ and P_2_S_7_^4–^ proceeds as described by Calpa et al. *via* reactions of P_4_S_12_^4–^ passing through P_2_S_6_^2–^,^21^ possibly involving the lithitropic tautomer proposed by Delnick et al.,^23^ which, however, has not been detected by us. It is important to underline that (PS_3_^–^)_*n*_ polymeric species does not appear to be the first intermediate species, as suggested before,^21^ but it forms only after P_4_S_11_^2–^.

## Conclusions

A deeper understanding of thiophosphate speciation in solution has been achieved through the investigation of the reaction between P_4_S_10_ and Li_2_S. This reaction unfolds in distinct intermediate stages, beginning with the solubilization of P_4_S_10_ and culminating in the formation of polymeric PS_3_^–^ species. SC-XRD data provide the first structural characterization of reaction intermediates that, until now, had been proposed. Although the ^31^P NMR spectroscopy previously enabled the assignment of chemical shifts to certain species, the ability to unambiguously identify these intermediates crystallographically offers crucial validation of the proposed reaction pathway through direct structural evidence.

Notably, this study provides the first direct spectroscopic and crystallographic evidence of the P_4_S_11_^2–^ anion, a key precursor to subsequent species such as P_2_S_6_^2–^, PS_4_^3–^, and P_2_S_7_^4–^. Alongside these, byproducts such as the two P_2_S_8_^2–^ isomers were also identified, highlighting the inherent complexity of thiophosphate chemistry in solution. While these investigations focused on the solution phase, solid-state techniques are planned to complement the findings by analyzing the solid precipitates, thus providing a more comprehensive picture of the reaction mechanisms involved.

As Masquelier et al. have emphasized,^15^ a significant challenge in the synthesis of solid electrolytes via liquid-phase methods is the inadvertent formation of species with lower ionic conductivity, such as P_2_S_6_^2–^. These species can adversely affect the overall conductivity of the electrolyte material, posing limitations for practical applications. However, this understanding also presents an opportunity: by carefully controlling the reaction conditions, it may be possible to influence the composition of the electrolytes and mitigate the formation of these less conductive byproducts. Such control could pave the way for enhancing the performance of batteries, underscoring the importance of continued research in this area.

## Supplementary Material



## Notes

NMR spectra and crystallographic data (PDF)
