# Supplementary material for: A Multinuclear NMR Study on the Speciation in the Liquid-Phase Synthesis of Sulfide-Based Electrolytes for All-Solid-State Lithium Batteries
Source: Inorg Chem. 2025 Aug 26;64(35):17754–63. doi: 10.1021/acs.inorgchem.5c02111 (PMC12421684; doi:10.1021/acs.inorgchem.5c02111)
Supplement: Supplementary file 1 [file ic5c02111_si_001.pdf]

SUPPORTING INFORMATION

A Multinuclear NMR Study on the Speciation in the Liquid-Phase Synthesis of Sulphides-Based Electrolytes for All-Solid-State Lithium Batteries

*Agnese Purgatorio,[a] Federico Ducale,[b] Leonardo Tensi,[c] Luca Rocchigiani,[a] Maurizio Leonardi,[b] and Alceo Macchioni [a]**

[a]Department of Chemistry, Biology and Biotechnology and CIRCC, University of Perugia, Via Elce di Sotto 8, 06123 Perugia, Italy

[b]Italmatch Chemicals S.p.A., Via S. Tommaso, 13, 06049 Spoleto (PG), Italy

[c]Department of Pharmaceutical Sciences, University of Perugia, Via del Liceo 1, 06123 Perugia, Italy

(*) alceo.macchioni@unipg.it

Supplementary NMR data

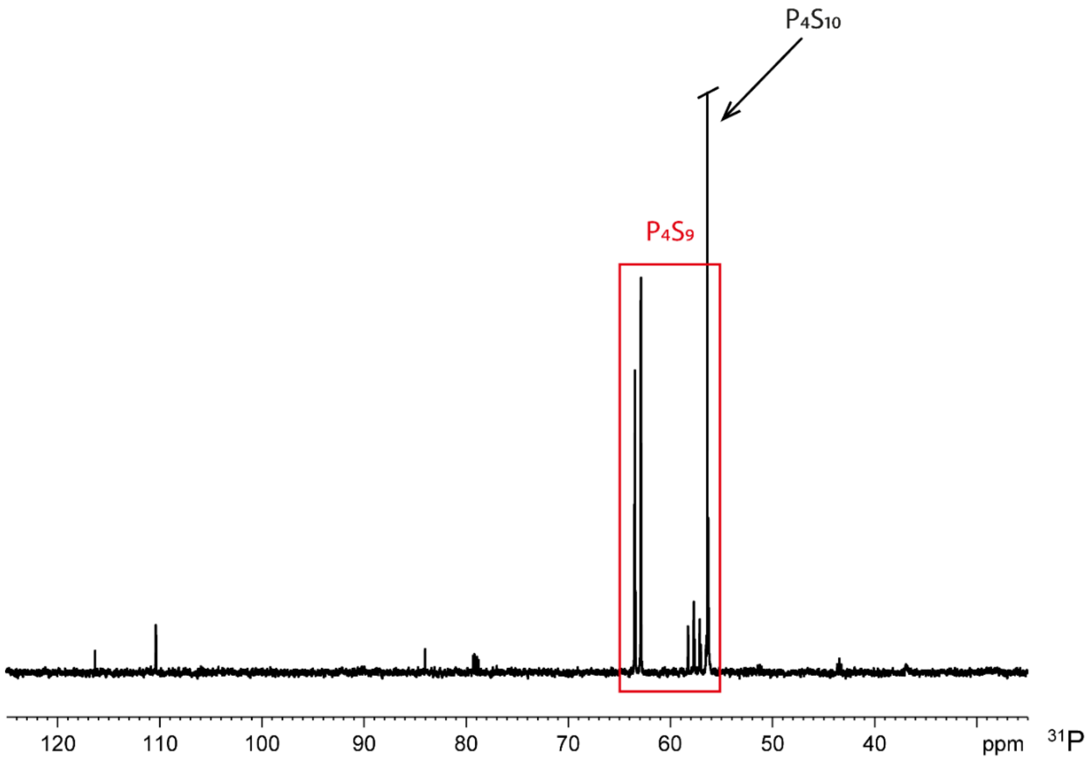

Figure S1. ^{31}P NMR spectrum of P_4S_{10} (400.13 MHz, CS_2/acetone-d_6 capillary, 298 K). Benzene was added as internal standard for the PGSE NMR measurements. δ = 63.0 – 56.1 (m, P_4S_9), 55.9 (s, P_4S_{10}).

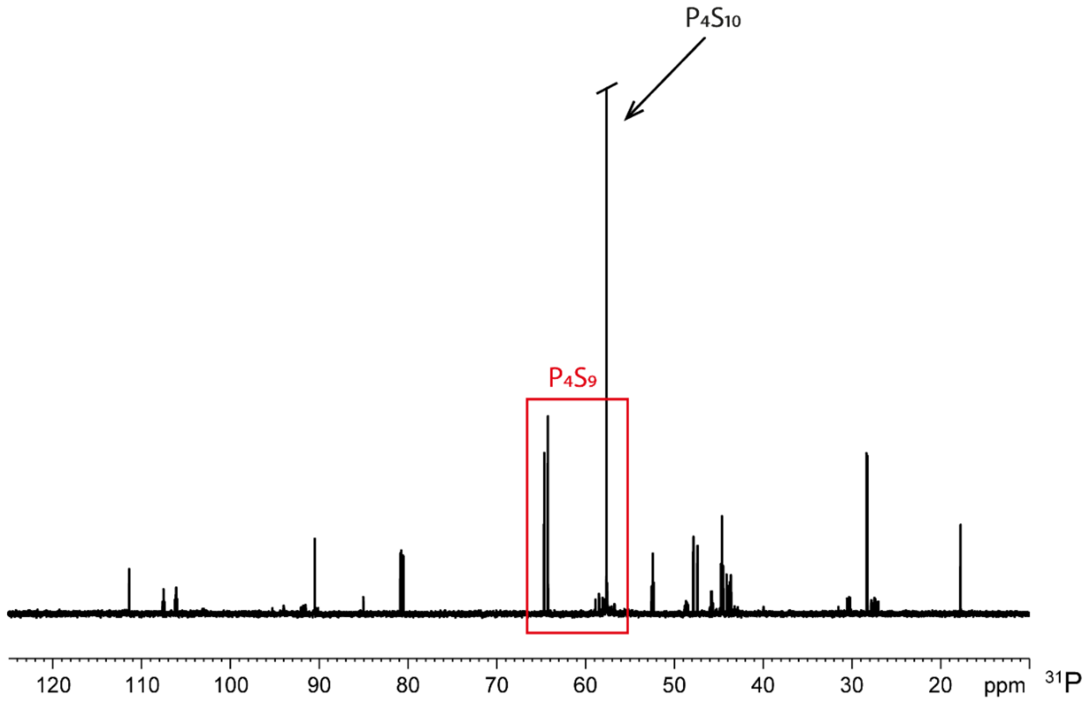

Figure S2. ^{31}P NMR spectrum of P_4S_{10} in toluene (600.13 MHz, toluene/acetone-d_6 capillary, 298 K). δ = 62.9 – 55.8 (m, P_4S_9), 55.9 (s, P_4S_{10}).

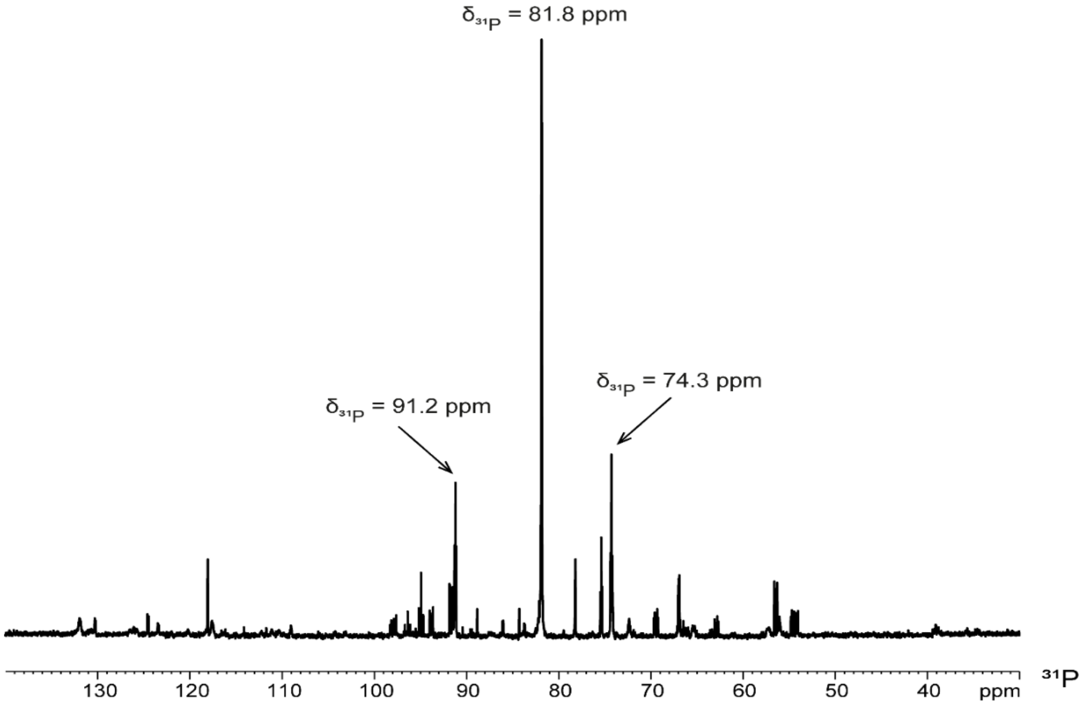

Figure S3. ^{31}P NMR spectrum of LiCl:P_4S_{10} = 1:1 molar ratio (400.13 MHz, acetonitrile-d_3, 298 K).

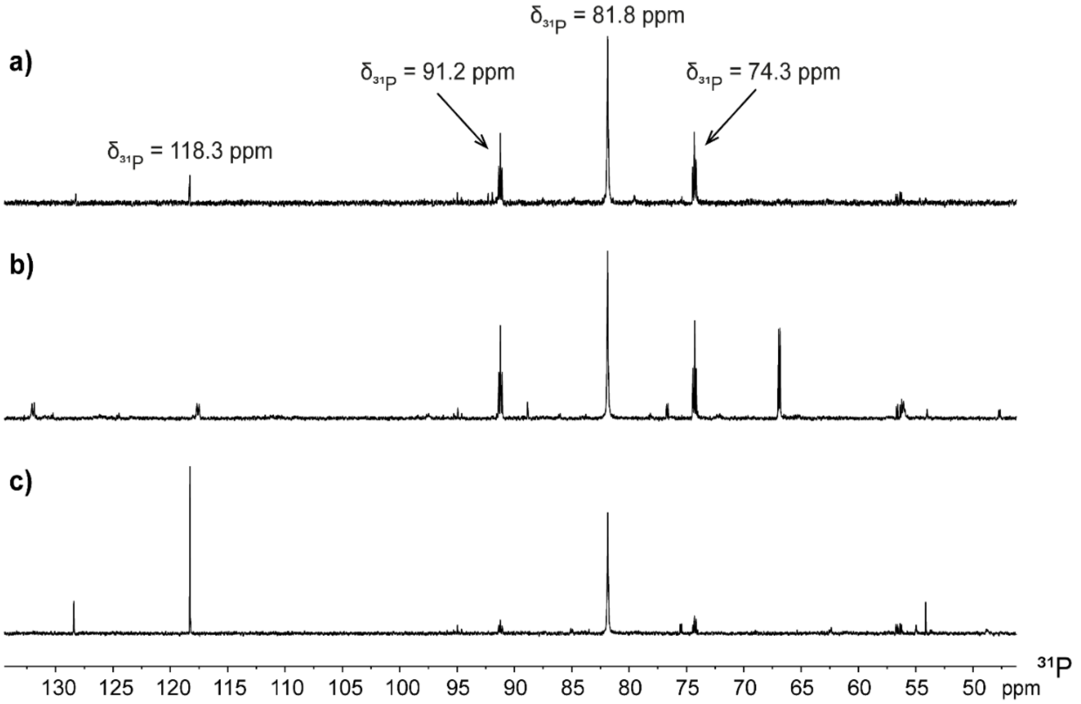

Figure S4. ^{31}P-NMR spectra of **a)** LiCl:P_4S_{10} = 1:1 molar ratio and **b)** 1 h and **c)** 15 days after addition of an excess of S_8 (400.13 MHz, acetonitrile-d_3, 298 K).

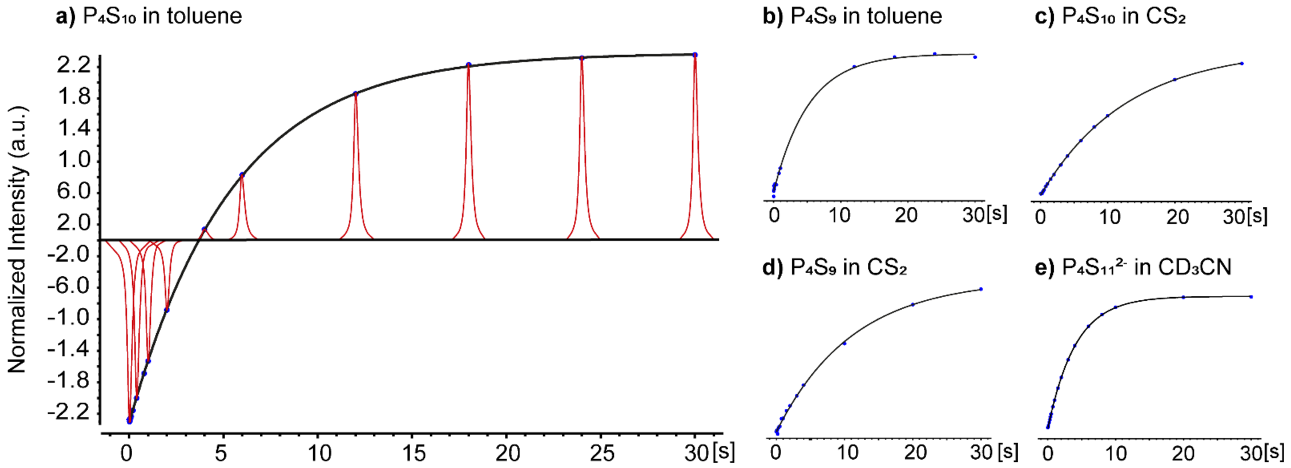

Figure S5. Intensity of the ^{31}P NMR resonance as a function of the recovery time for **a)** P_4S_{10} and **b)** P_4S_9 in toluene/acetone-d$_6$ capillary, **c)** P_4S_{10} and **d)** P_4S_9 in CS_2/acetone-d$_6$ capillary, and for **e)** $P_4S_{11}^{2-}$ in acetonitrile-d$_3$ (400.13 MHz, 298 K).

Ln(I/I$_0$) vs G^2 plots for the species studied. I = peak intensity, G = gradient strength in arbitrary units. Data were recorded in CS_2/acetone-d$_6$ capillary with benzene as internal standard (**Figure S6**), in toluene/acetone-d$_6$ capillary (**Figure S7**), and in CD_3CN (**Figures S8-S13**) at 298 K (400.13 MHz).

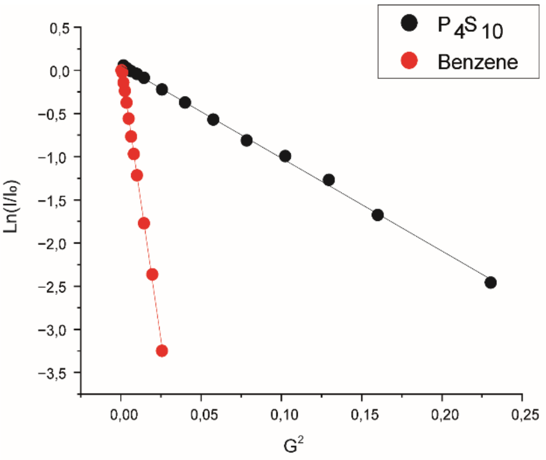

Figure S6

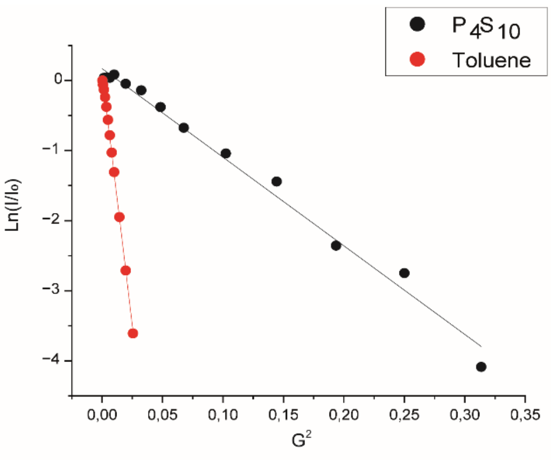

Figure S7

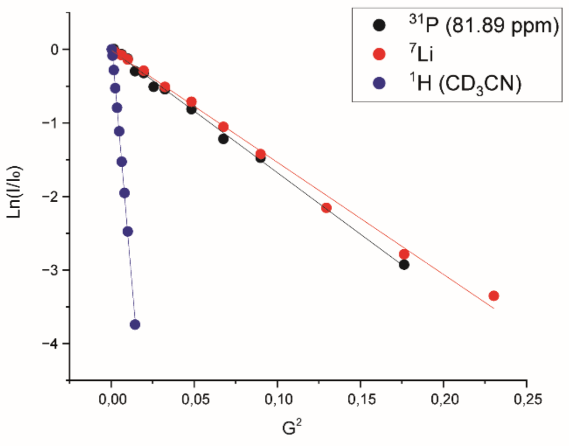

Figure S8. $Li_2S:P_4S_{10}$ = 0.125:1

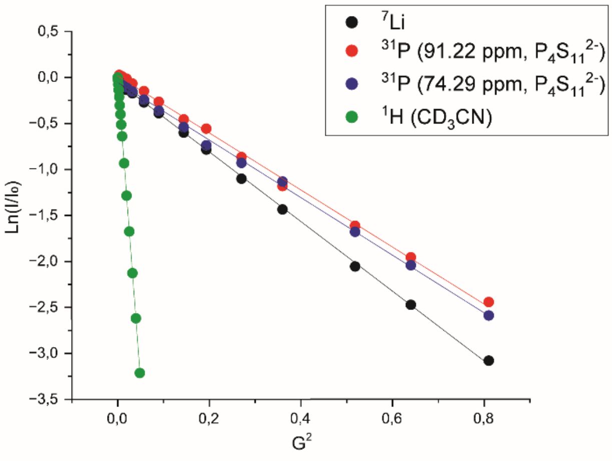

Figure S9. $Li_2S:P_4S_{10}$ = 0.25:1

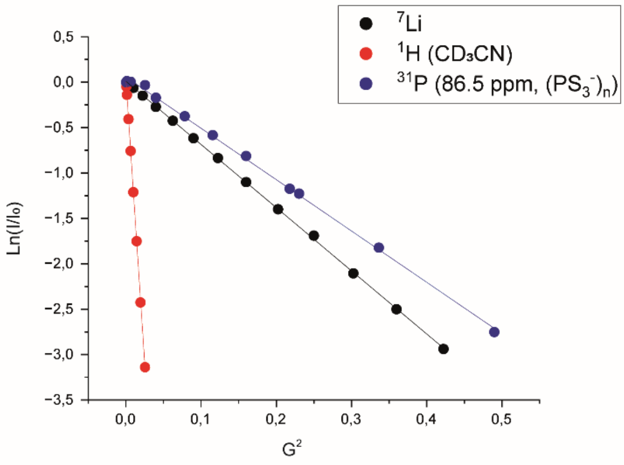

Figure S10. $Li_2S:P_4S_{10}$ = 2:1

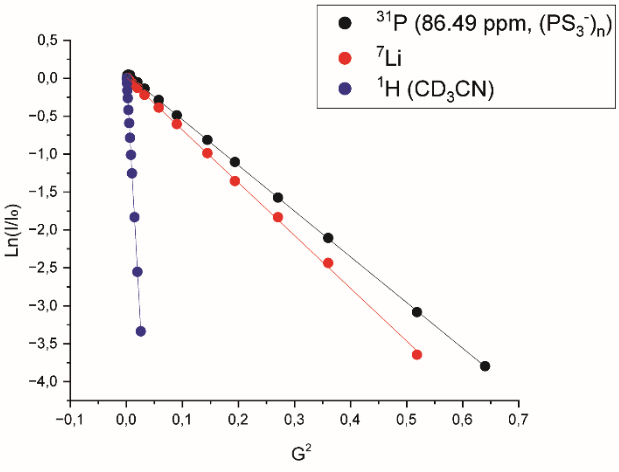

Figure S11. $Li_2S:P_4S_{10}$ = 4:1

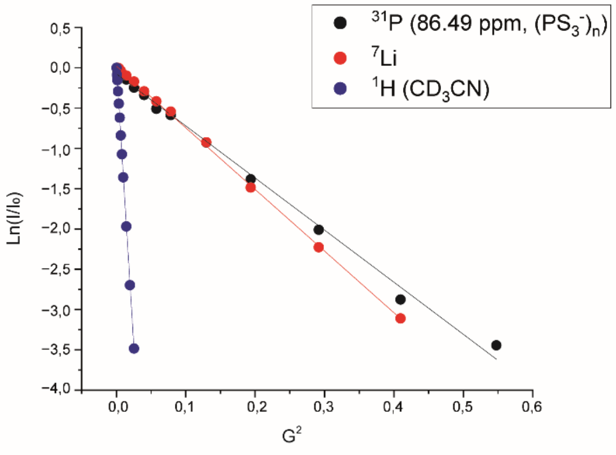

Figure S12. $Li_2S:P_4S_{10}$ = 14:3

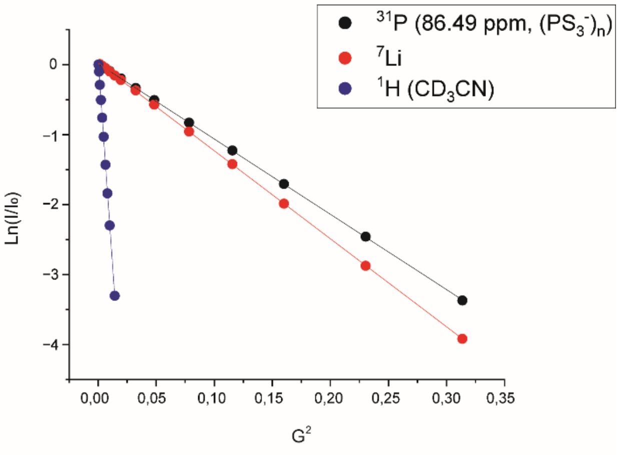

Figure S13. $Li_2S:P_4S_{10}$ = 6:1

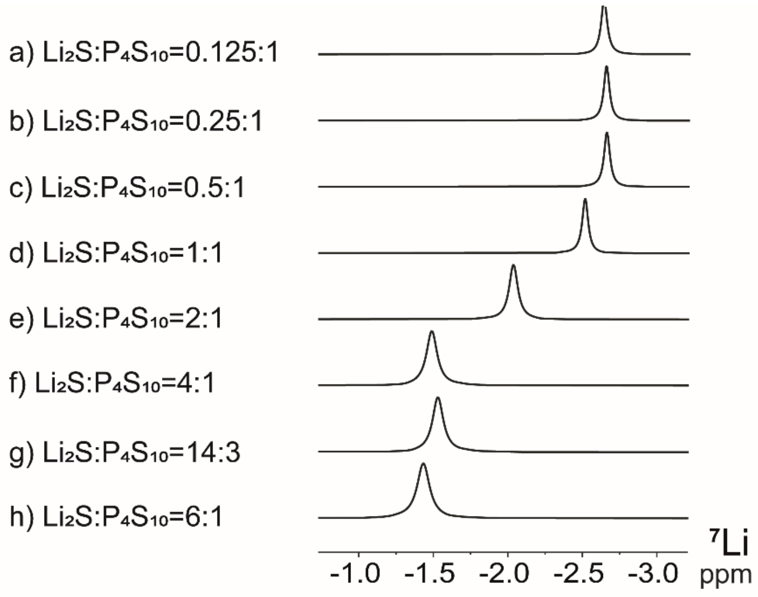

Figure S14. ^{7}Li NMR spectra at different $Li_2S:P_4S_{10}$ molar ratios (a-h, 600.13 MHz, acetonitrile-d_3, 298 K).

X-ray diffraction experiments

X-Ray diffraction single-crystal studies were performed using a Bruker D8 Venture diffractometer equipped with an Incoatec ImuS3.0 microfocus sealed-tube MoKα (λ = 0.71073 Å) source and a CCD Photon II detector. The analysis was carried out at low temperature using an Oxford Cryosystems Cryostream 800 cooler. The data, collected through generic φ and ω scans, were integrated and reduced using the Bruker AXS V8 Saint Software. The structures were solved, and all thermal parameters were anisotropically refined using the SHELXT and SHELXL packages of the Bruker APEX3 software.

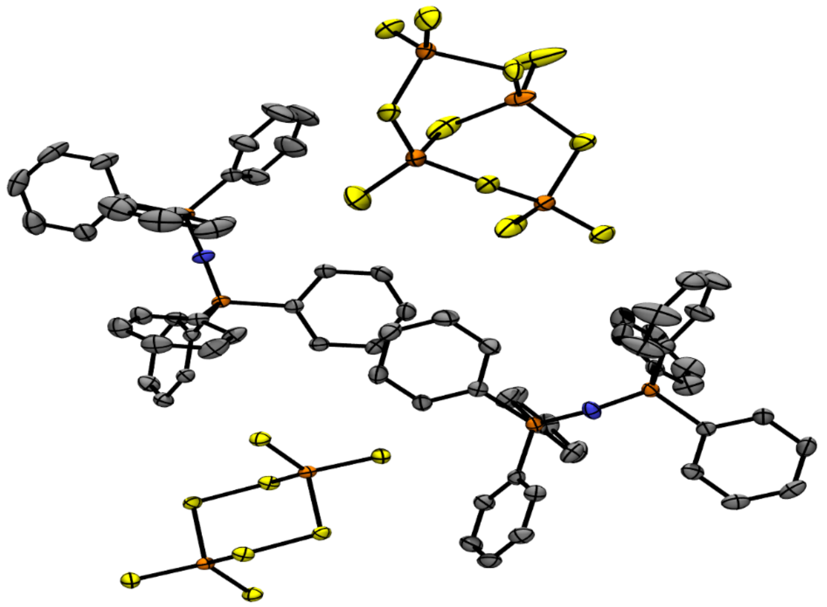

Figure S15. Ortep drawings of a part of the unit cell of the crystal **$[P_4S_{11}][PPN]_2$ + 0.9$[P_2S_8][PPN]_2$ + 0.1$[P_2S_6][PPN]_2$** (hydrogen atoms, PPN cations and additional $[P_2S_8]^{2-}$/$[P_2S_6]^{2-}$ anions are omitted for clarity). Ellipsoids are drawn at 50% probability level. Color code: P = Orange, S = Yellow, C = Gray, and N = blue. For further details see the table below.

Table S1. Crystallographic details of P_4S_{10} and $[P_4S_{11}][PPN]_2$ + $0.9[P_2S_8][PPN]_2$ + $0.1[P_2S_6][PPN]_2$.

	P_4S_{10}	$[P_4S_{11}][PPN]_2 + 0.9[P_2S_8][PPN]_2 + 0.1[P_2S_6][PPN]_2$
CCDC n.	2449521	2449522
Elemental formula	$P_4\ S_{10}$	$C_{72}\ H_{60}\ P_7\ S_{9.42}\ N_2$
Formula weight	444.48	1472.07
Crystal system	Triclinic	Triclinic
Space group	P-1	P-1
a = (Å)	9.0111(4)	10.8867(4)
b =	9.1484(4)	13.8734(5)
c =	9.1789(4)	23.4579(9)
α = (°)	92.532(2)	89.0370(10)
β =	101.044(2)	88.4050(10)
γ =	109.8980(2)	86.9920(10)
Volume ($Å^3$)	693.41(5)	3536.3(2)
Z, Calculated density (g/cm^3)	2, 2.129	2, 1.382
F(000)	440	1523
Absorption coefficient (mm^{-1})	2.007	0.497
Temperature (K)	150.(2)	150.(2)
Crystal colour, shape	Clear colourless, prism	Clear Yellow, prism
Crystal size (mm)	0.300 x 0.300 x 0.060	0.200 x 0.100 x 0.100
Theta range for data collection	2.2767 to 28.2837	2.5821 to 28.2508
Limiting indices	$-10\leq h\leq 12$, $-12\leq k\leq 12$, $-12\leq l\leq 12$	$-14\leq h\leq 14$, $-18\leq k\leq 16$, $-31\leq l\leq 31$
Completeness	96.5%	99.2%
Max. and min. transmission	0.7457 and 0.5993	0.7457 and 0.6940
Reflns collected (not incl. absences)	9606	51384
No. of unique reflns, R(int) for equivs	3328, 0.0198	17389, 0.0344
No. of 'observed' reflns ($I > 2\sigma_I$)	3080	14281
Data/restr./parameters	3328/0/127	17389/0/921
Goodness-of-fit on F^2	1.115	1.015
Final R indices ('obsd' data)	0.0184, 0.0451	0.0483, 0.1192
Final R indices (all data)	0.0206, 0.0464	0.0614, 0.1292
Reflns weighted: 1/w = [a]	[\s^2^(Fo^2^)+(0.0159P)^2^+0.2139P]	[\s^2^(Fo^2^)+(0.0527P)^2^+4.0022P]
Largest diff. peak and hole (e. $Å^{-3}$)	0.330 and -0.275	0.925 and -0.738

[a]where P=(Fo^2^+2Fc^2^)/3.

Table S2. Crystallographic details of $[P_2S_8][PPN]_2$ twisted and $[P_2S_6][PPN]_2$.

	$[P_2S_8][PPN]_2$ twisted	$[P_2S_6][PPN]_2$
CCDC n.	2449523	2449524
Elemental formula	C_{36} H_{30} P_3 S_4 N	C_{36} H_{30} N P_3 S_3
Formula weight	697.76	665.70
Crystal system	Monoclinic	Orthorombic
Space group	C2(1)/c	Pbca
a = (Å)	30.043(3)	15.9094(5)
b =	13.2136(14)	19.6346(8)
c =	20.5274(15)	20.9494(9)
α = (°)	90	90
β =	123.915(3)	90
γ =	90	90
Volume ($Å^3$)	6762.5(11)	6544.1(4)
Z, Calculated density (g/cm^3)	8, 1.371	8, 1.351
F(000)	2896	2768
Absorption coefficient (mm^{-1})	0.451	0.401
Temperature (K)	150.(2)	150.(2)
Crystal colour, shape	Clear Colourless, prism	Clear colourless, prism
Crystal size (mm)	0.100 x 0.060 x 0.060	0.800 x 0.100 x 0.020
Theta range for data collection	2.3589 to 27.0858	2.2910 to 28.2753
Limiting indices	$-39 \le h \le 39$, $-17 \le k \le 17$, $-24 \le l \le 27$	$-21 \le h \le 19$, $-22 \le k \le 26$, $-27 \le l \le 27$
Completeness	99.5%	99.9%
Max. and min. transmission	0.7457 and 0.6262	0.7457 and 0.65
Reflns collected (not incl. absences)	43877	85881
No. of unique reflns, R(int) for equivs	8353, 0.1316	8132, 0.0440
No. of 'observed' reflns ($I > 2\sigma_I$)	5031	6763
	Refinement	
Data/restr./param.	8353/87/422	8132/0/388
Goodness-of-fit on F^2	1.019	1.074
Final R indices ('obsd' data)	0.0620, 0.1085	0.0344, 0.0826
Final R indices (all data)	0.1085, 0.1344	0.0475, 0.0944
Reflns weighted: 1/w = [a]	[\s^2^(Fo^2^)+(0.0308P)^2^+20.4749P]	[\s^2^(Fo^2^)+(0.0348P)^2^+6.1189P]
Largest diff. peak and hole (e. $Å^{-3}$)	0.607 and -0.414	0.522 and -0.402

[a]where P=(Fo^2^+2Fc^2^)/3.
